# Circulating histone H3 levels are increased in septic mice in a neutrophil-dependent manner: preclinical evaluation of a novel sandwich ELISA for histone H3

**DOI:** 10.1186/s40560-018-0348-y

**Published:** 2018-11-26

**Authors:** Takashi Ito, Mayumi Nakahara, Yoshiki Masuda, Sachie Ono, Shingo Yamada, Hiroyasu Ishikura, Hitoshi Imaizumi, Chinatsu Kamikokuryo, Yasuyuki Kakihana, Ikuro Maruyama

**Affiliations:** 1 Department of Systems Biology in Thromboregulation, Kagoshima University Graduate School of Medical and Dental Sciences, Kagoshima, Japan; 2 Department of Emergency and Intensive Care Medicine, Kagoshima University Graduate School of Medical and Dental Sciences, Kagoshima, Japan; 3 Department of Anesthesiology and Critical Care Medicine, Kagoshima University Graduate School of Medical and Dental Sciences, Kagoshima, Japan; 4 Department of Intensive Care Medicine, Sapporo Medical University School of Medicine, Sapporo, Japan; 5 R&D Center, Shino-Test Corporation, Sagamihara, Japan; 6 Department of Emergency and Critical Care Medicine, Faculty of Medicine, Fukuoka University, Fukuoka, Japan; 7 Department of Anesthesiology and Intensive Care Medicine, Tokyo Medical University, Tokyo, Japan

**Keywords:** Histone, Sepsis, Neutrophil, ELISA

## Abstract

**Background:**

Nuclear histone proteins are released into the extracellular space during sepsis and act as major mediators of death. However, circulating histone levels have not been precisely quantified.

**Methods:**

We developed a novel enzyme-linked immunosorbent assay (ELISA) for detection of circulating histone H3 levels and evaluated its performance. Using the ELISA, we measured plasma histone H3 levels in C57BL/6 J mice subjected to cecal ligation and puncture (CLP)-induced sepsis.

**Results:**

The newly developed ELISA enabled reproducible measurement of histone H3 levels with a working range up to 250 ng/mL. Using the ELISA, we found that plasma histone H3 levels were elevated in septic mice compared with sham-operated mice (*p* < 0.01). The elevation of histone H3 levels was abrogated when neutrophils were depleted (*p* < 0.01).

**Conclusions:**

Our novel ELISA provides reproducible measurements of histone H3 levels. Circulating histone H3 levels are increased in septic mice in a neutrophil-dependent manner. Further studies are needed to evaluate the clinical utility of histone H3 levels in patients with sepsis.

## Background

Sepsis is a life-threatening disorder that results from dysregulation of the host response to infection [1, 2]. An overwhelming inflammatory response has been considered to be responsible for death from sepsis [3]. However, anti-inflammatory agents, such as tumor necrosis factor antagonists and interleukin-1 receptor antagonists, failed to improve the survival of patients with sepsis [3]. Molecules other than classical proinflammatory cytokines may be responsible for multiple organ failure and death in patients with sepsis.

Recent studies have suggested that damage-associated molecular patterns (DAMPs) may be possible mediators of death in sepsis [4, 5]. DAMPs are released from damaged cells, and enhance inflammation, coagulation, and bacteria killing in the extracellular milieu [6–9]. Among the DAMPs, extracellular histones are of particular concern because they are toxic toward host cells as well as bacteria, and can thus act as mediators of remote organ injury [5, 10, 11]. In addition to damaged cells, neutrophils can act as a source of extracellular histones in the form of neutrophil extracellular traps (NETs) [12–14]. Although NETs have a role in some antimicrobial innate immune responses, NETs and their fundamental histone components can induce organ damage. These observations have led to increased demands for a convenient and reproducible method that can measure circulating histone levels. However, the only method currently available for analysis of circulating histone levels is semiquantitative western blot analysis [5, 15, 16]. In this report, we describe a novel enzyme-linked immunosorbent assay (ELISA) for measurement of histone H3 levels in serum or plasma samples and its performance in preclinical settings.

## Materials and methods

### Measurement of histone H3 levels

Polystyrene microtiter plates (Nunc, Roskilde, Denmark) were coated with 100 μL/well of 1 mg/L anti-histone H3 peptide polyclonal antibody (Shino-Test Corporation, Sagamihara, Japan) in phosphate-buffered saline (PBS), and incubated overnight at 2–8 °C. After three washes with PBS containing 0.05% Tween-20, the remaining binding sites were blocked by incubation with 400 μL/well of PBS containing 1% bovine serum albumin for 2 h. The plates were washed again and incubated with 100 μL/well of diluted calibrator and serum samples (1:10 dilution in 0.2 mol/L Tris pH 8.5, 0.15 mol/L NaCl, and 1% bovine serum albumin) for 24 h at room temperature. After washing, the plates were incubated with 100 μL/well of anti-histone H3 peroxidase-conjugated peptide polyclonal antibody (Shino-Test Corporation) for 2 h at room temperature. The plates were washed again, and the chromogenic substrate 3,3′,5,5′-tetra-methylbenzidine (Dojindo Laboratories, Kumamoto, Japan) was added to each well. The reaction was terminated with 0.35 mol/L Na_2_SO_4_, and the absorbance at 450 nm was measured with a microplate reader (Model 680; Bio-Rad, Hercules, CA, USA). A standard curve was obtained with purified calf thymus histone H3 (Roche, Stockholm, Sweden). The amino acid sequence of histone H3 is highly conserved throughout species, and that of the antibody recognition site used in this ELISA completely matched between humans, calves, mice, and rats. This ELISA specifically detects histone H3 and does not react with other histone family proteins, including histone H2A, H2B, and H4, even if 10^4^ times excess proteins are loaded.

### Cecal ligation and puncture-induced sepsis in mice

All experiments involving animals were approved by the Institutional Animal Care and Use Committee of Kagoshima University. Male C57BL/6 J mice (Kyudo, Fukuoka, Japan) at 10–13 weeks of age were anesthetized with isoflurane and subjected to cecal ligation and puncture (CLP)-induced sepsis as described [17] with slight modifications. Briefly, the cecum of volatile-anesthetized mice was ligated below the ileocecal valve and punctured with a 21-gauge needle. A small amount of the intestinal content was extruded, and the cecum was relocated into the abdominal cavity. Sham mice underwent the same procedures, except for the cecal ligation and cecal puncture steps. After closing the abdominal wall, the mice were subcutaneously injected with 1 mL of normal saline to ameliorate hypovolemic shock. Subsequently, blood samples were collected from volatile-anesthetized mice at 6, 12, 24, and 36 h after CLP (*n* = 6–12 per time point) or sham operation (*n* = 4–10 per time point), and centrifuged at 1500×*g* for 10 min. Serum samples were stored at − 80 °C until further analysis.

### Leukocyte and neutrophil depletion in mice

For leukocyte depletion experiments, mice were injected intraperitoneally with 150 mg/kg and 100 mg/kg cyclophosphamide (Shionogi & Co. Ltd., Osaka, Japan) at 72 and 24 h prior to CLP, respectively. For neutrophil depletion experiments, mice were injected intravenously with 100 μg of anti-Ly-6G antibody (Bio X Cell, West Lebanon, NH) at 72 and 24 h prior to CLP. Leukocyte and neutrophil depletion was confirmed by complete blood counts obtained with an ADVIA120 (Siemens Healthcare Japan, Tokyo, Japan).

### Assessment of cellular damage

For assessment of cellular damage, the serum activities of cell-derived enzymes, including lactate dehydrogenase (LDH), aspartate transaminase (AST), and alanine transaminase (ALT), were examined in blood samples collected at 24 h after CLP or sham operation using a BioMajesty JCA-BM6070 (Jeol Ltd., Tokyo, Japan).

### Statistical analysis

Circulating histone H3 levels are shown as median (lower quartile–upper quartile) or box plot with lower extreme, lower quartile, median, upper quartile, and upper extreme values, as well as outliers. Differences in circulating histone H3 levels between CLP mice and sham mice were analyzed by the Mann–Whitney *U* test. Differences in leukocyte counts and serum enzyme activities between leukocyte/neutrophil-depleted mice and control mice were analyzed by Welch’s *t* test. A two-sided *p* value of less than 0.05 was considered statistically significant.

## Results

### Validation of the newly developed ELISA for circulating histone H3 levels

**Fig. 1:**
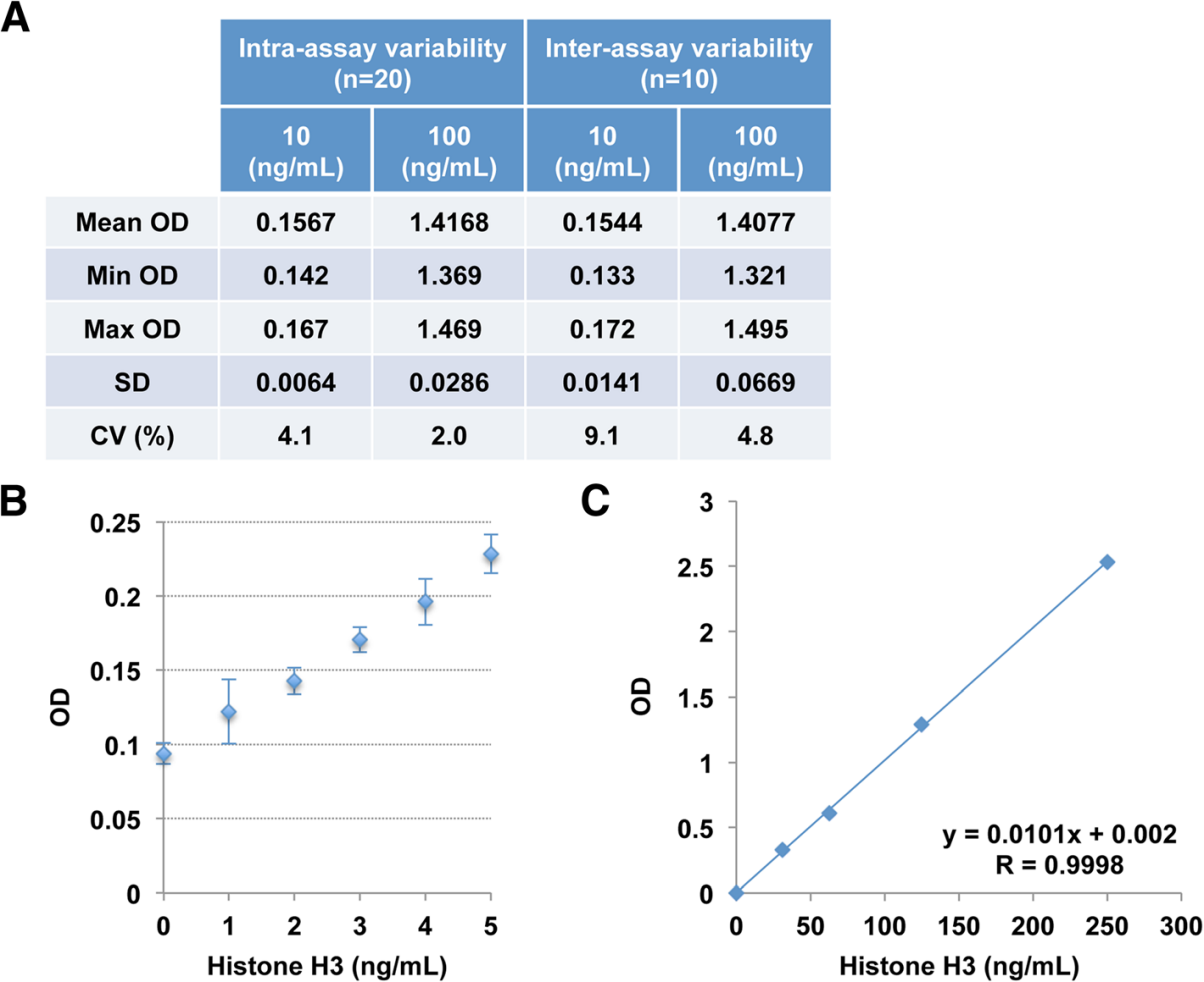
Validation of the newly developed ELISA for detection of circulating histone H3 levels. **a** Intra-assay and inter-assay precisions were analyzed using serum samples containing histone H3 at concentrations of 10 and 100 ng/mL. The intra-assay (*n* = 20) and inter-assay (*n* = 10) CVs were 2.0–4.1% and 4.8–9.1%, respectively. OD, optical density; CV, coefficient of variation. **b** The detection limit determined by the mean ± 2.6 SD method was 2 ng/mL. **c** Linearity was observed in the range up to 250 ng/mL

Initially, we developed a sandwich ELISA for histone H3 detection in serum and plasma samples and evaluated its performance. Purified calf histone H3 spiked into pooled serum samples was detected at 92–101% by the ELISA. Intra-assay and inter-assay precision was 2.0–4.1% and 4.8–9.1%, respectively (Fig. 1a). Accurate measurement of histone H3 was achieved at concentrations above 2 ng/mL (Fig. 1b). Linearity was observed in the range up to 250 ng/mL (Fig. 1c). Serum and plasma histone H3 levels did not differ in simultaneously collected samples, with some exceptions of samples containing relatively high serum histone H3 levels (data not shown). These results indicate that the newly developed ELISA provides reproducible measurements of circulating histone H3 levels in serum and plasma samples with a working range up to 250 ng/mL.

### Circulating histone H3 levels are elevated in mice with CLP

**Fig. 2:**
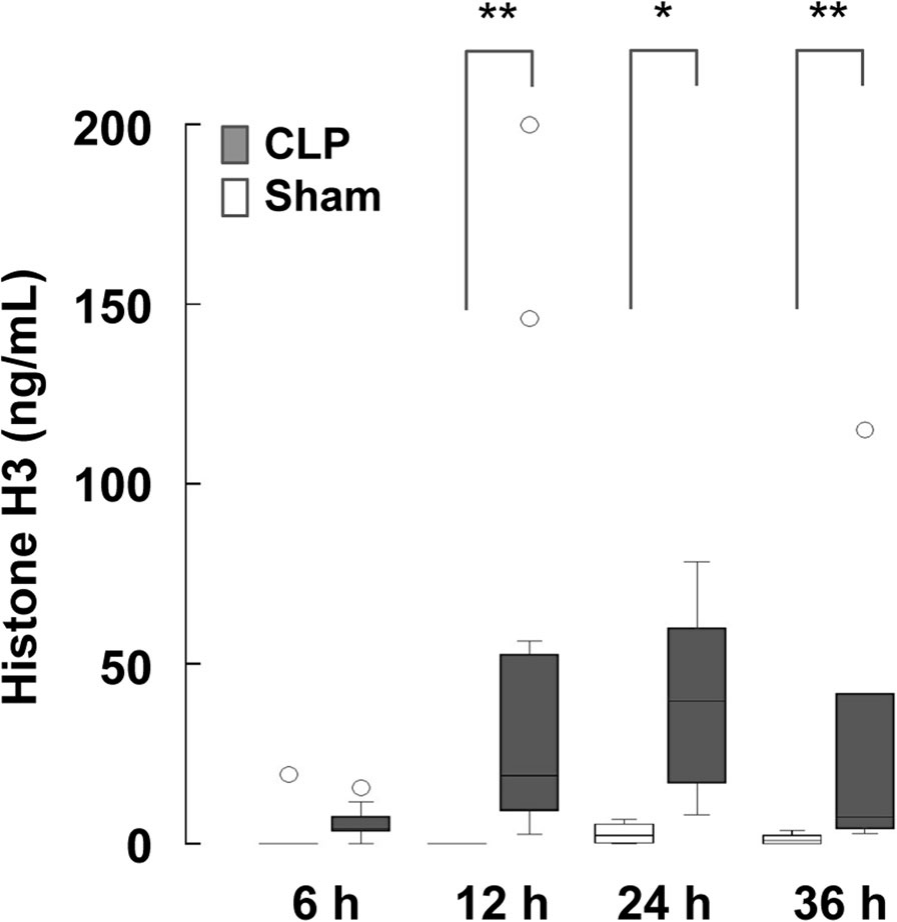
Circulating histone H3 levels are elevated in mice with CLP. Serum histone H3 levels in C57BL/6 J mice with CLP-induced sepsis or sham operation are shown. Serum samples were collected at 6, 12, 24, and 36 h after CLP (*n* = 6–12 per group) or sham operation (*n* = 4–10 per group). Representative data of two or more independent experiments are shown. Differences in circulating histone H3 levels between CLP mice and sham mice were analyzed by the Mann–Whitney *U* test. *p < 0.05, **p < 0.01

Using the ELISA, we examined the circulating histone H3 levels in mice subjected to CLP or sham operation. CLP is considered a realistic model for polymicrobial sepsis induction in experimental settings [17, 18]. CLP, but not sham operation, led to a significant increase in circulating histone H3 levels beginning at 12 h after surgery (Fig. 2). These results suggest that sepsis is a direct inducer of histone release into the systemic circulation.

### Circulating histone H3 is mainly derived from leukocytes under septic conditions

**Fig. 3:**
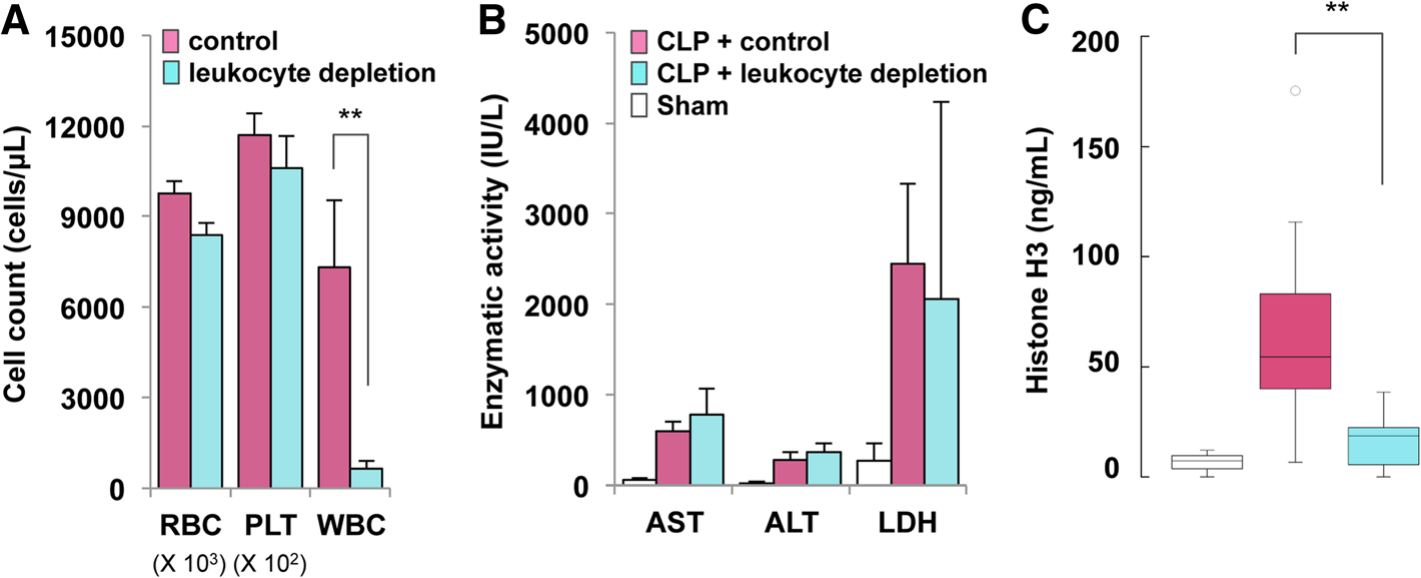
Circulating histone H3 levels are mainly derived from leukocytes in CLP mice. **a** Leukocyte depletion was achieved by intraperitoneal injection of 150 mg/kg and 100 mg/kg cyclophosphamide at 72 h and 24 h prior to blood cell counts, respectively. RBC, red blood cell (× 10^3^ cells/μL); PLT, platelet (× 10^2^ cells/μL); WBC, white blood cell (cells/μL). Differences in WBC counts between cyclophosphamide-injected mice (leukocyte depletion, *n* = 5, mean ± SD) and saline-injected mice (control, *n* = 6, mean ± SD) were analyzed by Welch’s *t*-test. **p < 0.01. **b** Leukocyte depletion was conducted by intraperitoneal injection of 150 mg/kg and 100 mg/kg cyclophosphamide at 72 and 24 h prior to CLP, respectively. For assessment of cellular damage, the activities of AST, ALT, and LDH at 24 h after CLP or sham operation were examined. The AST, ALT, and LDH levels at 24 h after CLP did not differ significantly between leukocyte-depleted mice (*n* = 9, mean ± SD) and control mice (*n* = 10, mean ± SD). **c** Serum histone H3 levels at 24 h after CLP were examined in leukocyte-depleted mice (*n* = 9) and control mice (*n* = 10). Representative data of two independent experiments are shown. Differences in circulating histone H3 levels were analyzed by the Mann–Whitney *U* test. **p < 0.01

The cellular sources of circulating histone H3 levels remain unclear. Leukocytes are a likely source because they comprise the majority of nucleated cells in the blood [9]. Another potential source is damaged tissues, wherein high rates of apoptosis or necrosis overwhelm the phagocytic system, thereby allowing histone H3 to enter the circulation [19]. To investigate the possible involvement of leukocytes in histone release under septic conditions, we examined circulating histone H3 levels in leukopenic mice subjected to CLP. Leukocyte depletion was achieved by intraperitoneal injection of cyclophosphamide (Fig. 3a). Tissue damage, shown by elevation of AST, ALT, and LDH levels, was comparable between leukopenic mice and control mice at 24 h after CLP (Fig. 3b). However, circulating histone H3 levels were significantly lower in leukopenic mice compared with control mice (Fig. 3c). These findings indicate that circulating histone H3 levels under septic conditions are mainly derived from leukocytes and not from damaged cells.

### Circulating histone H3 is mainly derived from neutrophils under septic conditions

**Fig. 4:**
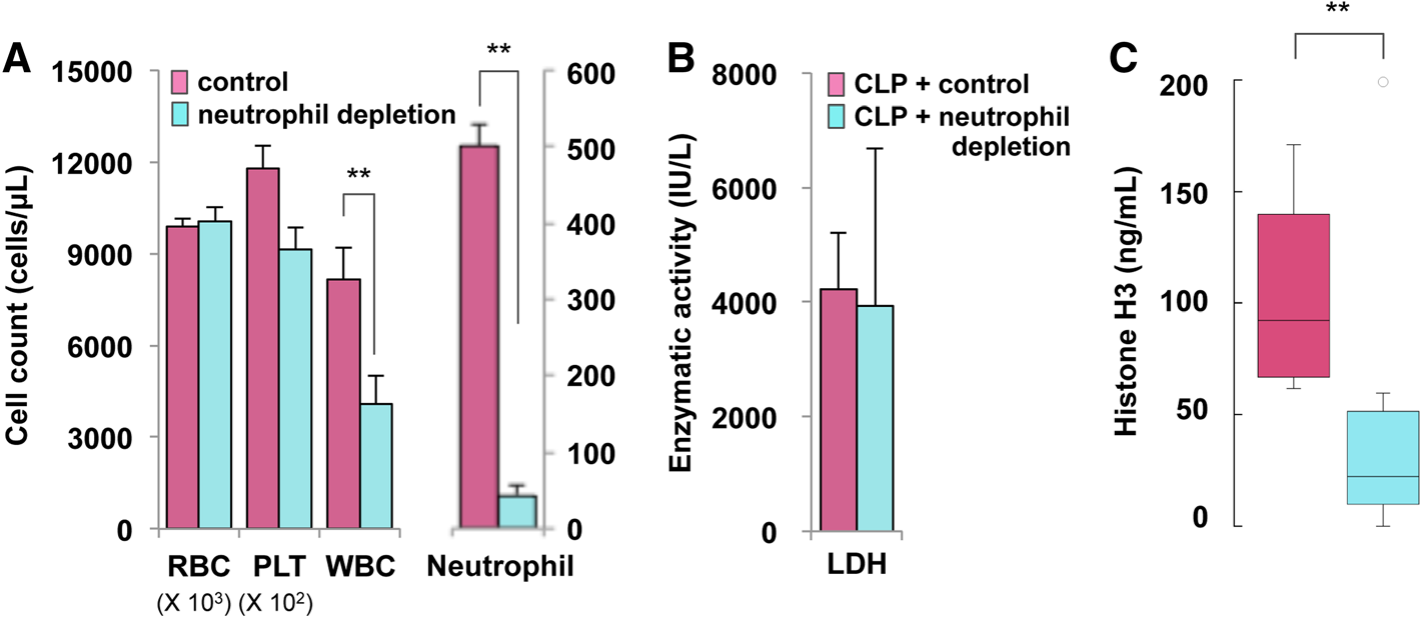
Circulating histone H3 levels are mainly derived from neutrophils in CLP mice. **a** Neutrophil depletion was conducted by intravenous injection of 100 μg/mouse of anti-Ly-6G antibody at 72 and 24 h prior to blood cell counts. RBC, red blood cell (× 10^3^ cells/μL); PLT, platelet (× 10^2^ cells/μL); WBC, white blood cell (cells/μL). Differences in white blood cell (WBC) and neutrophil counts between anti-Ly-6G antibody-injected mice (neutrophil depletion, *n* = 8, mean ± SD) and isotype control antibody-injected mice (control, *n* = 5, mean ± SD) were analyzed by Welch’s *t* test. **p < 0.01. **b** Neutrophil depletion was achieved by intravenous injection of 100 μg/mouse of anti-Ly-6G antibody at 72 and 24 h prior to CLP. For assessment of cellular damage, the serum LDH levels at 24 h after CLP were examined. The serum LDH levels at 24 h after CLP did not differ significantly between neutrophil-depleted mice (*n* = 6, mean ± SD) and control mice (*n* = 10, mean ± SD). **c** Serum histone H3 levels at 24 h after CLP were examined in neutrophil-depleted mice (*n* = 8) and control mice (*n* = 10). Representative data of two independent experiments are shown. Differences in circulating histone H3 levels were analyzed by the Mann–Whitney *U* test. **p < 0.01

Among leukocytes, neutrophils are the most likely source of circulating histones because they release histones extracellularly in the form of NETs [12, 13]. Therefore, we examined the circulating histone H3 levels in neutropenic mice subjected to CLP. Neutrophil depletion was achieved by intravenous injection of an anti-Ly-6G antibody (Fig. 4a). Tissue damage was comparable between neutropenic mice and control mice (Fig. 4b). However, circulating histone H3 levels were significantly lower in neutropenic mice compared with control mice at 24 h after CLP (Fig. 4c). These findings indicate that circulating histone H3 levels are mainly derived from neutrophils under septic conditions.

## Discussion

In this study, we demonstrated that our newly developed ELISA provided reproducible measurements of circulating histone H3 levels. Plasma histone H3 levels in septic mice were higher than those in non-septic mice. Furthermore, the circulating histone H3 levels under septic conditions were mainly derived from neutrophils, and not from damaged cells in multiple organs. Under septic conditions, neutrophils release histones extracellularly in the form of NETs [12, 13]. NETs, which are web-like structures of decondensed DNA complexed with antimicrobial histones, neutrophil elastase, and myeloperoxidase, can entrap and kill microbes in the extracellular milieu [20]. In response to microbial stimuli, neutrophils initiate a program involving citrullination of histones, rearrangement of nuclear and granular architectures, and extracellular release of cellular contents in the form of NETs. This is an active process in viable neutrophils, rather than a passive release of cellular contents from dead neutrophils [21]. It remained unclear whether the circulating histone H3 detected by our ELISA was a component of NETs. Specific detection of citrullinated histone H3 or simultaneous detection of histone H3 and neutrophil granule proteins may help to understand the forms of histone H3 in the systemic circulation.

Circulating histone H3 levels were significantly lower in septic mice with leukopenia compared to those with normal leukocyte count; however, these levels were not as low as non-septic mice. These findings suggest that cells other than leukocytes may also contribute to circulating histone H3 levels. Damaged cells are a possible source because dying renal cells and necrotic hepatocytes are known to release histones into the extracellular space during sterile inflammation [10, 22]. On the other hand, it is also reported that histones remain anchored to the insoluble chromatin of necrotic cells and thus may not be released into the extracellular space [23]. Further studies are needed to ensure that histones can be released from damaged cells.

In this study, circulating histone H3 levels were slightly increased at 6 h and markedly increased at 12 h after CLP. This time course suggests that histones are a kind of late phase mediator rather than an early biomarker of sepsis. Considering that circulating histones are toxic toward host cells and act as mediators of remote organ injury [5, 10, 11], measurement of histone H3 levels may be important in identifying severely ill patients who need intensive therapy rather than identifying patients in the early phase sepsis.

The present study has several limitations. First, the absolute values of circulating histone H3 levels remain controversial. Previous studies have described circulating histone H3 levels in orders of magnitude from picogram per liter [10] to microgram per milliliter [15]. In this study, the circulating histone H3 levels were in the nanogram per milliliter order of magnitude. This discrepancy among studies may be due to the heterogeneous composition of circulating histone H3 levels in vivo. In mammalian nucleated cells, a tetramer of histones H3 and H4 and two dimers of histones H2A and H2B forms a core particle around which 147 bp of DNA is wrapped 1.67 times [24]. When histone H3 is released into the extracellular space, it can exist in a complex with these proteins and various lengths of DNA or in a free form. The composition of the complex and free form of histone H3 in plasma can differ in individual cases, and the affinity for each form can differ among measurement methods. This may create discrepancies in the absolute values of circulating histone levels between measurement methods. Further studies are required for uncovering the condition of circulating histone H3. Second, a limitation of the CLP-induced sepsis model in mice should also be taken into account. Although this model is frequently used to investigate the pathophysiological mechanisms of sepsis, the metabolic and cardiovascular profiles of CLP-induced sepsis in mice are different from those of sepsis in humans [25]. Caution should be applied when extrapolating the findings in septic mice to septic patients. Finally, circulating histone H3 levels in diseases other than sepsis have not been examined in this study. Previous studies have suggested that these levels could be increased in non-septic diseases, such as trauma, pancreatitis, and ischemia/reperfusion injury [10, 11, 22]. These findings suggest that histone-related diseases are not limited to sepsis but include sterile inflammatory diseases.

## Conclusions

We have developed a novel sandwich ELISA that is suitable for measuring large numbers of samples and providing reproducible measurements of circulating histone H3 levels. Circulating histone H3 levels were increased in septic mice, and neutrophils were the major source of the circulating histone H3.

## Abbreviations

ALT: Alanine transaminase
AST: Aspartate transaminase
CLP: Cecal ligation and puncture
CV: Coefficient of variation
DAMPs: Damage-associated molecular patterns
ELISA: Enzyme-linked immunosorbent assay
LDH: Lactate dehydrogenase
NETs: Neutrophil extracellular traps
OD: Optical density
PBS: Phosphate-buffered saline
PLT: Platelet
RBC: Red blood cell
WBC: White blood cell (leukocyte)
